# Combining SARS-CoV-2 Proofreading Exonuclease and RNA-Dependent RNA Polymerase Inhibitors as a Strategy to Combat COVID-19: A High-Throughput *in silico* Screening

**DOI:** 10.3389/fmicb.2021.647693

**Published:** 2021-07-20

**Authors:** Shradha Khater, Pawan Kumar, Nandini Dasgupta, Gautam Das, Shashikant Ray, Amresh Prakash

**Affiliations:** ^1^Department of Biosciences and Bioengineering, Indian Institute of Technology Bombay, Mumbai, India; ^2^miBiome Therapeutics LLP, Mumbai, India; ^3^National Institute of Immunology, New Delhi, India; ^4^Department of Biotechnology, Mahatma Gandhi Central University, Motihari, India; ^5^Amity Institute of Integrative Sciences and Health, Amity University Haryana, Gurgaon, India

**Keywords:** SARS-CoV-2, drug repurposing, exonuclease inhibitor, nucleoside analog, molecular docking and MD simulation, combinatorial therapy

## Abstract

Severe acute respiratory syndrome coronavirus 2 (SARS-CoV-2) has infected millions of people worldwide. Currently, many clinical trials in search of effective COVID-19 drugs are underway. Viral RNA-dependent RNA polymerase (RdRp) remains the target of choice for prophylactic or curative treatment of COVID-19. Nucleoside analogs are the most promising RdRp inhibitors and have shown effectiveness *in vitro*, as well as in clinical settings. One limitation of such RdRp inhibitors is the removal of incorporated nucleoside analogs by SARS-CoV-2 exonuclease (ExoN). Thus, ExoN proofreading activity accomplishes resistance to many of the RdRp inhibitors. We hypothesize that in the absence of highly efficient antivirals to treat COVID-19, combinatorial drug therapy with RdRp and ExoN inhibitors will be a promising strategy to combat the disease. To repurpose drugs for COVID-19 treatment, 10,397 conformers of 2,240 approved drugs were screened against the ExoN domain of nsp14 using AutoDock VINA. The molecular docking approach and detailed study of interactions helped us to identify dexamethasone metasulfobenzoate, conivaptan, hesperidin, and glycyrrhizic acid as potential inhibitors of ExoN activity. The results were further confirmed using molecular dynamics (MD) simulations and molecular mechanics combined with generalized Born model and solvent accessibility method (MM-GBSA) calculations. Furthermore, the binding free energy of conivaptan and hesperidin, estimated using MM-GBSA, was −85.86 ± 0.68 and 119.07 ± 0.69 kcal/mol, respectively. Based on docking, MD simulations and known antiviral activities, and conivaptan and hesperidin were identified as potential SARS-CoV-2 ExoN inhibitors. We recommend further investigation of this combinational therapy using RdRp inhibitors with a repurposed ExoN inhibitor as a potential COVID-19 treatment.

## Introduction

**FIGURE 1:**
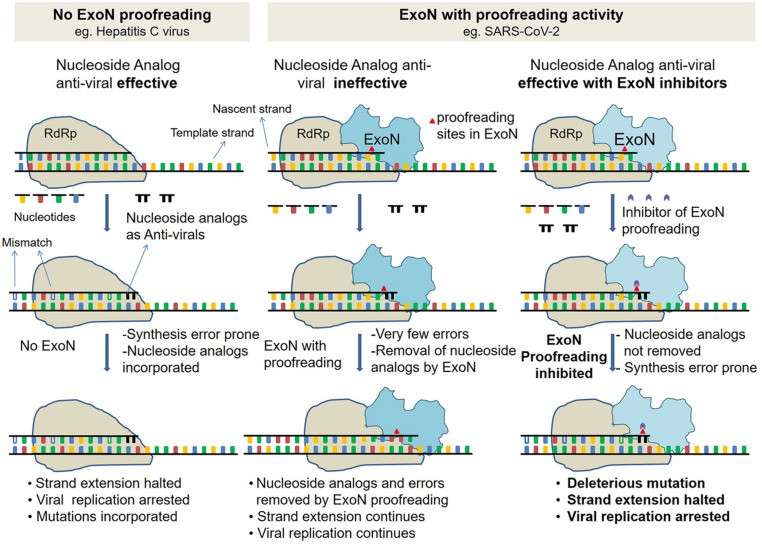
Schematic describing ExoN proofreading activity and mode of action of inhibitors. (Left) Replication in viruses such as hepatitis C virus (HCV), with no proofreading mechanism. Replication by low fidelity RNA-dependent RNA polymerase (RdRp) in absence of ExoN is error-prone. Incorporated nucleoside analogs (NAs) are not excised, resulting in either premature replication termination or incorporation of mutation. (Middle) Replication in viruses such as SARS-CoV with proofreading exonuclease (ExoN). Like HCV, in SARS-CoV-2 too, replication is by low-fidelity RdRp, but errors and NA are excised by proofreading ExoN, and decreasing the efficacy of antivirals. (Right) Same as middle panel but in presence of ExoN inhibitor. In presence of ExoN inhibitor, NA might not be excised, and resulting in premature replication termination.

On December 31, 2019, the World Health Organization (WHO) office in China was informed that cases of pneumonia of an unknown cause were detected in Wuhan City, in the Hubei Province of China. The Chinese authorities identified this to be a previously unknown type of coronavirus, severe acute respiratory syndrome coronavirus 2 (SARS-CoV-2), and causing the disease COVID-19. Since the outbreak, the number of confirmed cases of COVID-19 increased rapidly, resulting in WHO declaring the disease a pandemic on March 11, 2020 (Cucinotta and Vanelli, 2020). Almost all countries are affected by this pandemic with millions of confirmed cases and more than a million deaths worldwide. Currently, there are no approved drugs. However, there is an urgent need to have a repertoire of repurposed drugs to improve the efficacy and in addition be prepared for drug resistance. Synergistic action of a combination of drugs against SARS-CoV-2 can enhance the effectiveness of existing drugs that have shown partial success in clinical trials. The class of antivirals that are RNA-dependent RNA polymerase (RdRp) inhibitors, such as favipiravir, remdesivir, ribavirin, and galidesivir, has been on high priority since the beginning of COVID-19 trials. Trials have been completed or are in progress in many countries. Among these, remdesivir and favipiravir have shown promise in different countries. These drugs being nucleoside analogs act either by introducing mutations in the viral RNA or by chain termination during replication. The action of these drugs on viruses that do not have proofreading enzymes is good (Warren et al., 2016). However, SARS-CoV-2 possesses a nonstructural protein nsp14, with amino-terminal domain coding for a proofreading exonuclease (ExoN) (Bouvet et al., 2012). ExoN is capable of excising incorporated nucleoside analogs by virtue of its 3′–5′ exonuclease proofreading activity. This results in negating the action of these drugs, to varying extents, and depending on the type of nucleoside analog chemistry [ribavirin, 5-fluorouracil (5FU), and remdesivir] (Figure 1; Smith et al., 2013; Ferron et al., 2018). Hence, in the case of repurposed drugs for COVID-19, a limitation of efficacy exists. The new-generation RdRp inhibitors, such as remdesivir, are more effective than ribavirin and 5FU, and as excision of these nucleosides by viruses harboring exoribonuclease is weaker than ribavirin and 5FU (Ogando et al., 2019). The delicate balance between incorporation and excision properties of nucleoside analogs by RdRp and ExoN respectively, decides the fate of the action of RdRp-based antivirals.

Exonuclease inactivation was found to confer a “mutator phenotype,” as was evident from a 15- to 21-fold increase in mutation frequency—relative to the wild-type control—during replication and passaging in cell culture (Eckerle et al., 2007, 2010). In ExoN mutant background, remdesivir has 4.5-fold (Shannon et al., 2020), and ribavirin has 200-fold higher efficacy (Ferron et al., 2018), compared to a wild-type ExoN viral genome. The mutagenesis results are supported by sequencing analyses too (Eckerle et al., 2010). This provides a clear rationale to use a combination of antivirals favipiravir/remdesivir/ribavirin/galidesivir and SARS-CoV-2 ExoN inhibitors. Currently, there is no drug available to inhibit ExoN. Detailed molecular docking studies to find small molecules/peptides/natural molecules that have the potential to inhibit ExoN are urgently required (Senanayake, 2020). It is interesting to note that coronaviruses lacking ExoN are susceptible to lethal mutagenesis (Smith et al., 2013). The crystal structure of SARS-CoV nsp14-nsp10 (Ma et al., 2015) provides opportunities for molecular docking of the ExoN domain of nsp14 to different available drugs.

In this study, we propose that combinatorial therapy with one drug from favipiravir/remdesivir/ribavirin/galidesivir and an inhibitor of ExoN would be effective in increasing the efficacy of the RdRp inhibitors (Figure 1). To repurpose drugs for COVID-19 treatments, we performed molecular docking of 10,397 approved drug conformers on the ExoN domain of SARS-CoV-2 nsp14. Three known antivirals conivaptan, hesperidin, and glycyrrhizic acid show promise based on the docking results and their known inhibitory effects on β-coronaviruses *in vitro* (De Clercq, 2006) and in patients (Hoever et al., 2005; Ledford, 2020). Further docked complexes of conivaptan, hesperidin, glycyrrhizic acid, and astemizole were refined using 200-ns-long MD simulations. Binding energy estimation using molecular mechanics combined with generalized Born model and solvent accessibility method (MM-GBSA) studies estimated binding free energies of conivaptan and hesperidin as −85.86 ± 0.68 and 119.07 ± 0.69 kcal/mol, respectively. Therefore, repurposing hesperidin and conivaptan as potential inhibitors of proofreading ExoN and using them in conjunction with RdRp inhibitors could lead to a potentially high level of antiviral activity and promising therapy for COVID-19.

## Results

### SARS-CoV-2 ExoN Domain

SARS-CoV-2 nsp14 is a multidomain protein. The N-terminal domain functions as proofreading exoribonuclease, and the C-terminal is a methyltransferase. SARS-CoV-2 nsp14 shares 95.07% amino acid sequence identity (over complete protein length) with SARS-CoV nsp14 (Supplementary Figure 1). ExoN domain of SARS-CoV nsp14 resembles DEDD-type ExoNs (Ma et al., 2015). The DEDD superfamily members are defined by the presence of three canonical motifs—DXE (motif I), W(X)4EL (motif II), and DAIMTR (motif III) (Shannon et al., 2020). The presence of DEED instead of DEDD and an additional H makes the SARS-CoV ExoN a DEEDh-type ExoN (Ogando et al., 2019). In SARS-CoV-2, the catalytic residues—Asp90, Glu92, Glu191, His268, and Asp273, and the canonical motifs are conserved (Supplementary Figure 1). A 3-dimensional (3D) model of SARS-CoV-2 nsp14 was built using SARS-CoV nsp14 (PDB ID: 5C8S) as a template. A grid comprising the three conserved motifs was used for docking.

### Molecular Docking

**FIGURE 2:**
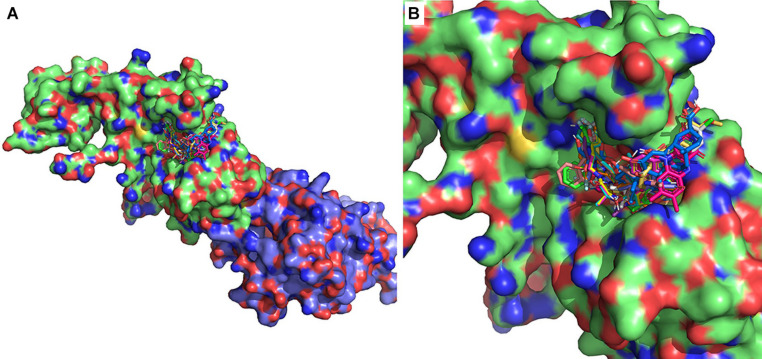
Twenty lowest-binding energy conformations from the molecular screen. **(A)** SARS-CoV-2 nsp14 is depicted as surface representation and the 20 lowest-binding energy poses are depicted as sticks. The ExoN domain is in green, and MTase domain is in blue. **(B)** Zoomed-in version depicting bound conformers of drug molecules.

**FIGURE 3:**
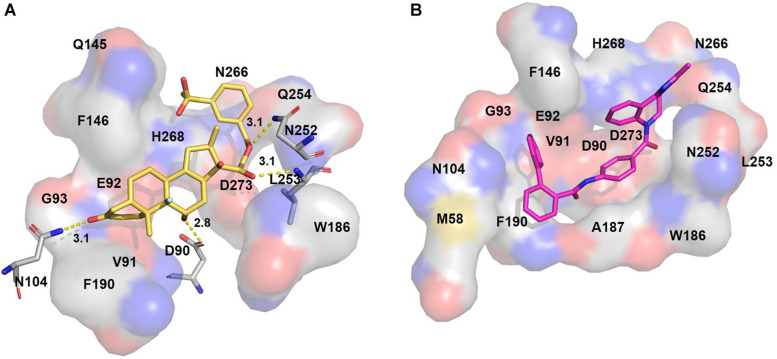
Putative binding pockets of docked compounds. Interaction of ExoN residues (gray) with docked conformer of dexamethasone metasulfobenzoate (yellow sticks) **(A)** and conivaptan (pink sticks) **(B)**. H-bonded residues are depicted as sticks. Yellow dotted lines represent hydrogen bond, and the distance is in angstroms (Å). Residues in hydrophobic contact are depicted as surface representation.

**FIGURE 4:**
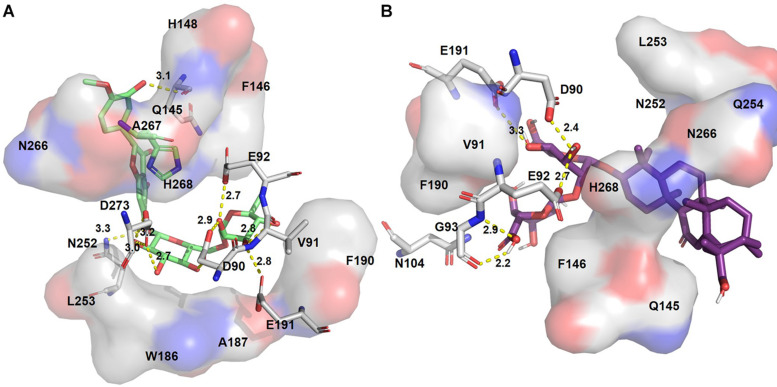
Putative binding pockets of docked compounds. Interaction of ExoN residues (gray) with docked conformer of hesperidin (green sticks) **(A)** and glycyrrhizic acid (purple sticks) **(B)**. H-bonded residues are depicted as sticks. Yellow dotted lines represent hydrogen bond, and the distance is in angstroms (Å). Residues in hydrophobic contact are depicted as surface representation.

Ten thousand three hundred ninety-seven conformers generated from 2,240 approved small molecule drugs were screened using AutoDock VINA. Based on binding free energy, the top 20 binding poses were selected for further analysis (Figure 2 and Table 1). All 20 poses interact with catalytic residues. Dexamethasone metasulfobenzoate binds to the catalytic site of ExoN with the binding energy of −8.7 kcal/mol. Conivaptan, dutasteride, hesperidin, lumacaftor, and glycyrrhizic acid bind ExoN active site with the slightly higher energy of −8.6 kcal/mol. Interaction of ExoN domain with 12 unique drug molecules, corresponding to top 20 poses, was studied and is depicted in Table 2. Most of the analyzed poses interact with at least three of the five catalytic residues (Figures 3, 4).

**TABLE 1:** Screening results of top twenty conformers with lowest-binding energies.

Drug bank ID	Conf ID	Name	Binding free energy (kcal/mol)
DB14703	1	Dexamethasone metasulfobenzoate	−8.7
DB00872	3	Conivaptan	−8.6
DB01126	1	Dutasteride	−8.6
DB01126	3	Dutasteride	−8.6
DB04703	1	Hesperidin	−8.6
DB09280	1	Lumacaftor	−8.6
DB13751	3	Glycyrrhizic acid	−8.6
DB14703	0	Dexamethasone metasulfobenzoate	−8.6
DB14703	2	Dexamethasone metasulfobenzoate	−8.6
DB14703	3	Dexamethasone metasulfobenzoate	−8.6
DB14703	4	Dexamethasone metasulfobenzoate	−8.6
DB00696	2	Ergotamine	−8.5
DB01126	0	Dutasteride	−8.5
DB03147	3	Flavin adenine dinucleotide (FAD)	−8.5
DB06210	1	Eltrombopag	−8.5
DB00637	3	Astemizole	−8.4
DB00696	1	Ergotamine	−8.4
DB00696	3	Ergotamine	−8.4
DB00878	0	Chlorhexidine	−8.4
DB01251	1	Gliquidone	−8.4

**TABLE 2:**
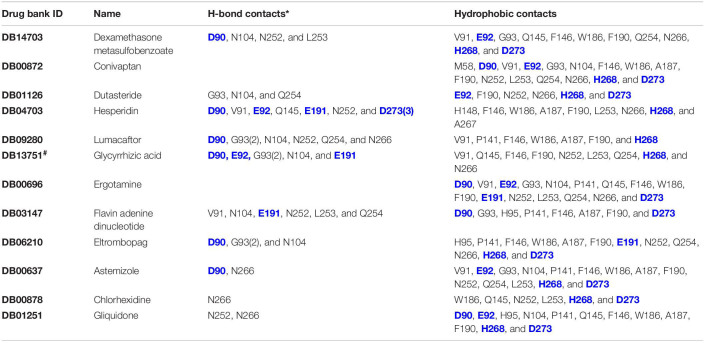
Residues involved in hydrogen bond and hydrophobic interaction.

Blue and bold font indicates catalytic residues. *Numbers in parentheses indicate the number of hydrogen bonds formed with the particular amino acid. ^#^LigPlus did not work, manually done.

Hesperidin is the only drug that interacts with all five catalytic residues. Hesperidin and glycyrrhizic acid have four and three ExoN catalytic residues, respectively, within hydrogen-bonding distance (Figure 4). The binding of these drugs to ExoN catalytic residues might potentially cause obstruction of substrate binding and catalysis.

Remdesivir, an investigational drug for the treatment of Ebola, was shown to inhibit SARS-CoV-2 RdRp, and inhibiting RNA synthesis (Gordon et al., 2020). As remdesivir is a drug without anti-ExoN activity, it was used here as a negative control. Remdesivir displayed a low binding affinity toward ExoN (−6.0 kcal/mol) than the top 20 poses (<−8.4 kcal/mol). The estimated binding energy of remdesivir with ExoN is -6.0 kcal/mol, higher than the top 20 poses from the approved drug category (Table 1).

### Structural Stability of ExoN and ExoN–Drug–Bound Complexes

Molecular dynamics simulation can provide atomistic insights on structural stability and the dynamic of protein–ligand interactions (Luthra et al., 2009; Prakash and Luthra, 2012; Wang et al., 2013; Panda et al., 2020). Based on molecular docking, interactions with active site ExoN residues and their antiviral properties, conivaptan, hesperidin, and glycyrrhizic acid were chosen for MD studies. As astemizole was shown to inhibit SARS-CoV-2 in *in vitro* assays, it was included in the MD studies (Riva et al., 2020).

**FIGURE 5:**
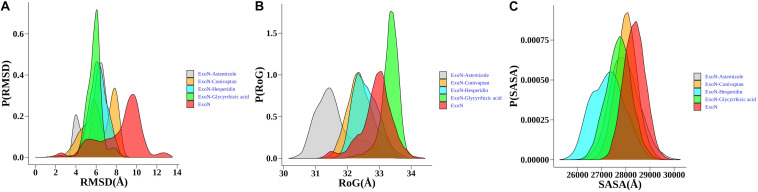
Probability distribution plots of structural order parameters. **(A)** C^α^ -backbone RMSD, **(B)** RoG, **(C)** SASA of ExoN, the docked complexes, ExoN–astemizole and ExoN–conivaptan, ExoN–hesperidin, and ExoN–glycyrrhizic acid.

The structural dynamics of glycyrrhizic acid, astemizole, conivaptan, and hesperidin in complex with ExoN displays maximum population density of stable conformation at ∼6.0, 6.5, 8, and 6 Å, respectively, relative to ExoN, which equilibrated at around 9.75 Å. Hence, drug molecules induced substantial rigidification in ExoN structure (Figure 5A). ExoN–glycyrrhizic acid exhibited the least structural fluctuations, suggesting the most stable protein–ligand complex. Although the complex of ExoN–conivaptan achieved a maximum population density of around 8 Å, the population density of conformational dynamics ranges from ∼4.0 to 9 Å. The ExoN–conivaptan complex shows a slightly smaller peak at ∼6.0 Å too. It suggests conivaptan might move between two conformations. The structure of ExoN and ExoN–glycyrrhizic acid, astemizole, conivaptan, and hesperidin had a maximum population density of radius of gyration (RoG) around 33, 33.5, 31.5, 32.2, and 32.2 Å, respectively (Figure 5B). During the simulation period of 200 ns, all five systems were stable around the solvent-accessible surface area (SASA) values of 2,700 to 2,900 Å^2^. RoG and SASA results suggest marginal or no structural compactness change of ExoN and ExoN–drug complexes (Figure 5C).

To understand the drifts in root mean square deviation (RMSD) plots (Figure 5 and Supplementary Figure 2A), the average distance of the four drug molecules from the center of the ExoN active site was measured. The time evolution distance plots show that the average distance of hesperidin and conivaptan remained consistent between 3.5 and 4.5 Å from the active site of ExoN (Supplementary Figure 3). Glycyrrhizic acid and astemizole move out from the binding pocket around ∼50 and ∼100 ns of simulation, respectively. The conformational adaptability of hesperidin and conivaptan during the simulation was explored by performing root mean square fluctuation (RMSF) analyses. The average RMSF peaks of all the amino acids of ExoN-hesperidin and ExoN–conivaptan complex are less than ExoN (Supplementary Figure 2 and Supplementary Methods). The RMSF values provide structural evidence of stable molecular interaction of hesperidin and conivaptan with ExoN. The average distances between metal ions (Mg^2+^) remain around 3.6 Å (Supplementary Figures 3, 4).

### Hydrogen Bond Analysis

The efficacy of a drug molecule is largely dependent on molecular interactions at the active site (Schiebel et al., 2018; Mishra et al., 2021), and the network of H-bonds plays a crucial role in this interaction. Thus, H-bond interactions between ExoN and drug molecules were calculated (Chen et al., 2016). Distance cutoff of 3.5 Å, and angle cutoff of 135° were used for the calculation of H-bonds. Maximum occupancy of seven H-bonds between ExoN and hesperidin was observed, of which five to six H-bonds were observed consistently during the simulation (Supplementary Figure 6). The molecular interaction of conivaptan with ExoN shows the maximum possibility of two H-bonds. Out of that, only one H-bond remains consistent throughout the simulation time (0–200 ns).

### Binding Free Energy Analysis MM-GBSA

To ascertain the molecular binding interaction of hesperidin and conivaptan with ExoN, a quantitative assessment of binding free energy (Δ*G*_*binding*_) was carried out using MM-GBSA (Genheden and Ryde, 2015) on the conformational ensemble of protein–ligand complexes. Hesperidin shows more favorable binding free energy, Δ*G*_*binding*_ = −119.07 ± 0.69 kcal/mol, as compared to conivaptan (Δ*G*_*binding*_ = −85.86 ± 0.68 kcal/mol) (Table 3).

**TABLE 3:** Binding free energy (kcal/mol) calculation of drug molecules against ExoN.

Compound	Δ*G*_*binding*_	Δ*E*_*vdW*_	Δ*E*_*electrostatic*_	Δ*E*_*GB*_	Δ*E*_*SURF*_	Δ*G*_*gas*_	Δ*G*_*solv*_
Conivaptan	−85.86 ± 0.68	−27.59 ± 0.31	−54.58 ± 0.67	0.56 ± 0.45	−4.25 ± 0.02	−82.17 ± 0.70	−3.69 ± 0.43
Hesperidin	−119.07 ± 0.69	−48.21 ± 0.49	−102.29 ± 0.84	38.49 ± 0.59	−7.05 ± 0.02	−150.51 ± 0.83	31.43 ± 0.58

**FIGURE 6:**
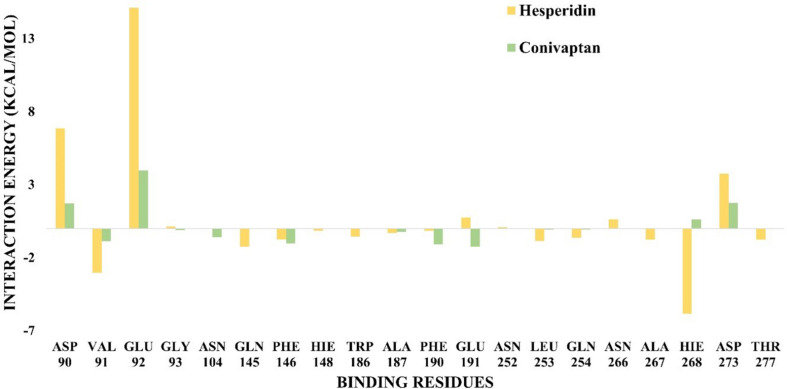
Binding free energy decomposition plot. Decomposition plot of binding free energy over the interacting residues at the active site of ExoN.

Free energy decomposition per residue at ExoN active site indicates energetically favorable molecular binding of hesperidin and conivaptan, largely contributed by the residues involved in van der Waals and electrostatic interactions (Figure 6). Hesperidin shows energetically favorable binding to catalytic residues Val91 and Hie268 and other active site residues Trp186, Ala187, Asn252, Leu253, Gln254, and Asn266. Interactions of these residues with hesperidin were observed in the molecular docking studies too (Figure 4A). Conivaptan interacts with catalytic residues Phe146, Phe190, and Glu191 and active site residues Val91, Gln145, Phe146, Trp186, Ala187, and Phe190. Like hesperidin, the interaction of the aforementioned residues with conivaptan was observed in molecular docking (Figure 4B).

## Discussion

COVID-19, an infectious respiratory illness, is caused by a novel strain of coronavirus SARS-CoV-2. Currently, there are no approved drugs. Drugs targeting RdRp such as favipiravir, remdesivir, ribavirin, and galidesivir have shown promise in SARS-CoV-2 and few other strains of coronavirus. Nucleoside analogs—remdesivir and favipiravir—have been authorized for emergency use in the treatment of COVID-19 in different countries. These RdRp inhibitors act by competing with host nucleoside substrates for incorporation in nascent RNA being synthesized (Xu et al., 2003). The misincorporated analogs cause either a premature chain termination or mutation in RNA (Figure 1). ExoN domain of nsp14 in CoVs is known to excise misincorporated analogs (Ferron et al., 2018). The excision action of ExoN decreases the efficacy of nucleoside analog, such as ribavirin, *in vivo* (Ferron et al., 2018). A decrease in efficacy of remdesivir was speculated because of ExoN activity (Gordon et al., 2020). A 100-fold increase in remdesivir efficacy was seen in ExoN mutant of murine hepatitis virus (MHV), a beta-coronavirus (Agostini et al., 2018). It has been hypothesized that simultaneous inhibition of RdRp and ExoN in CoVs could be an effective therapeutic strategy (Pruijssers and Denison, 2019).

Hence, *in silico* drug screening method was used to search for potential inhibitors of ExoN. Ten thousand three hundred ninety-seven conformers from 2,240 approved drugs were screened against SARS-CoV-2 nsp14 containing the ExoN domain. AutoDock VINA screening results indicated dexamethasone metasulfobenzoate to be the top binder and conivaptan, dutasteride, hesperidin, lumacaftor, and glycyrrhizic acid to be a close second. All six compounds showed extensive interaction with nsp14 residues, especially the catalytic residues of the ExoN domain (Table 2).

Interestingly, few of the approved drugs that bind with ExoN catalytic site *in silico*– conivaptan, hesperidin, and glycyrrhizic acid—have shown antiviral activity in earlier studies. Conivaptan, a nonpeptide inhibitor of vasopressin, has shown *in vitro* efficacy against feline infectious peritonitis coronavirus, human coronavirus OC43 (HCoV-OC43), dengue, and Zika virus (Yang et al., 2020). Inhibitory activity of hesperidin against influenza a virus was reported by Dong et al. (2014). Its antivirus response was linked to increases in cell-autonomous immune responses (enhanced expression of primary and secondary genes). In addition, hesperidin inhibited the export of viral ribonucleoproteins. Hesperitin, an aglycone form of hesperidin, was shown to inhibit the cleavage activity of SARS-CoV 3C–like protease (Lin et al., 2005). Glycyrrhizic acid, an antitumoral, anti-inflammatory drug, has *in vitro* inhibitory effects on a broad range of viruses like flaviviruses (Crance et al., 2003), herpesviruses, and human immunodeficiency virus (Lin, 2003). Glycyrrhizic acid was used for the treatment of SARS-CoV (Hoever et al., 2005) and chronic hepatitis virus in patients (Miyake et al., 2002). Astemizole was reported to inhibit SARS-CoV-2 *in vitro* assays (Riva et al., 2020). Based on molecular docking results and varying degrees of evidence in support of their antiviral use, conivaptan, hesperidin, glycyrrhizic acid, and astemizole were selected for MD studies.

Dexamethasone, our top hit in docking screen, is a glucocorticoid shown to reduce fatality by a third in critically ill COVID-19 patients requiring ventilator support (Ledford, 2020). Glucocorticoids are known to cause adverse effects and are not recommended for use in mild COVID-19 cases. Hence, the use of dexamethasone as a potential ExoN inhibitor was not pursued further.

In-depth MD studies revealed that spatial orientation of hesperidin and conivaptan favors stable molecular interaction, and they remain well occupied at the ExoN active site for the entire duration of the simulation (Figure 5 and Supplementary Figure 3). Binding free energy calculations using MM-GBSA indicated a higher binding affinity of hesperidin compared to conivaptan. Although the binding affinity of conivaptan was lower than hesperidin, both the drugs are involved in energetically favorable molecular interactions with catalytic and active site residues (Figure 6 and Table 3). Hence, we hypothesize that glycyrrhizic acid and conivaptan might occlude ExoN catalytic site, thereby inhibiting the proofreading activity.

Our docking result, MD simulations combined with evidence in support of antiviral use of glycyrrhizic acid and conivaptan, and underscores their potential as SARS-CoV-2 ExoN inhibitor. When used in combination with RdRp inhibitors, the higher concentrations reported for the repurposed ExoN inhibitors to exert antiviral activity could be minimized. In addition, the higher concentrations required for RdRp inhibitors to exert their action in the presence of ExoN activity such as in SARS-CoV-2 can also be reduced, as dual action of RdRp and ExoN inhibition in parallel should require lower concentrations of the respective drugs. A combination of RdRp and ExoN inhibitors, in addition to the increased efficacy, would also possibly avert drug resistance generated from mutations in ExoN to enhance the proofreading of nucleoside analogs in RdRp inhibition.

## Materials and Methods

### Homology Modeling of ExoN

The amino acid sequence of SARS-CoV-2 ExoN/nsp14 (P0DTD1) was used as a target sequence to build a 3D model using the SWISS-MODEL web server (Schwede et al., 2003). Nsp14 from SARS-CoV (PDB ID: 5C8S, chain B) had a high sequence identity to target sequence—95.07%. This 3D structure was used as a template for building the SARS-CoV-2 nsp14 model. The model quality was assessed using the QMEAN score. The QMEAN score for the generated model was −3.14. Scores greater than −4 indicate a good model.

### Virtual Screening Using AutoDock VINA

Two-dImensional structures of approved small molecule drugs were downloaded from DrugBank in sdf format (2,454 structures) (Wishart et al., 2006). The structures were converted to 3D format using the OpenBabel –gen3d option (O’Boyle et al., 2011; Yoshikawa and Hutchison, 2019). Few of the drug molecule structures showed error at this stage. 3D structures of these molecules were downloaded from other sources (Supplementary Table 1). Multiple conformers for each 3D structure were generated using OpenBabel to increase the conformational space sampling.

The docking grid was defined to encompass the conserved motives of the ExoN domain (see section “SARS-CoV-2 ExoN Domain”). A total of 10,397 conformers from 2,240 compounds were subjected to virtual screening using the AutoDock VINA tool on SARS-CoV-2 nsp14 (Trott and Olson, 2010). Twenty poses were generated for each compound. Compound-nsp14 interactions were visualized using LigPlot^+^ v2.1 (Laskowski and Swindells, 2011).

### Modeling of Mg^2+^ Ion in the Active Site

SARS-CoV-2 ExoN crystal structure (PDB ID: 7MC6, length 291 amino acids) became available after the completion of our work. Pairwise structure comparison of SARS-CoV-2 ExoN crystal structure and homology model (this study) was done using the Dali web server (Holm, 2020). The comparison revealed the two structures to be highly similar (DALI *Z* score 39.7; RMSD 0.8 over 285 aligned residues).

The SARS-CoV-2 structure contains one Mg^2+^ ion in the catalytic site (Moeller et al., 2021). ExoN uses two metal ions to remove misincorporated nucleotides. In the structures of SARS-CoV-2 and SARS-CoV, ExoN coordinates of only one Mg^2+^ ion are observed. Lassa virus NP ExoN, an ExoN of DEDDh-family, contains two Mn^2+^ ions in its catalytic site. The two Mg^2+^ ions were modeled in the docked complexes based on the Mn2+ ion position in the Lassa NP ExoN-RNA complex (PDB ID: 4GV9). Simulations were performed using the Mg^2+^ ions containing docked structures.

### MD Simulation

All-atoms MD simulation was performed using the Amber16 with force field ff14SB (Case et al., 2005; Maier et al., 2015) for the metal ion–containing protein, ExoN, and the docked complexes with drug molecules, astemizole, conivaptan, hesperidin, and glycyrrhizic acid. Antechamber (Wang et al., 2006) is used to parameterize all selected ligands using the GAFFs force field (Wang et al., 2004). Divalent Mg^++^ interactions are modeled using the 12-6-4 model compatible with the TIP3P water model. For the ligand preparation, topology, and parameter files were generated using the leap module of Amber (Maier et al., 2015). Keeping the protein at the center, a cubic box is prepared with 10 Å, padding the explicit TIP3P water molecules in all directions (Jorgensen et al., 1983), and the counter-ions (Na^+^Cl^–^) added to neutralize the simulation box. Particle mesh Ewald approach (Essmann et al., 1995) was used for electrostatic interaction calculation and the SHAKE algorithm (Ryckaert et al., 1977) was used to constrain H-bonds. Energy minimization of prepared systems was performed in three stages, each of 10,000 steps of steepest descent (SD) and conjugate gradient (CG) to relax the system. Furthermore, each simulation system was gradually heated from 50 to 300 K in six steps, followed by 10,000 steps of SD and CG minimization, respectively. Under the NVT ensemble condition, each system is equilibrated for 1 ns. Finally, all five systems were submitted for the production run under NPT ensemble condition for 200 ns with a time step of 2 fs.

### MD Trajectory Analysis and MM-GBSA Assay

The obtained MD trajectories were analyzed for the structural stability of ExoN and binding with drug molecules through the RMSD, RMSF, RoG, SASA, and H-bond interactions between protein and ligands during the simulation. The Cα-backbone RMSD is calculated with reference to the starting structure of the protein. RMSF defines the average positional fluctuations of the protein residues from their initial position, which is important to determine the local dynamics of a protein. The binding free energy for each complex drug molecule was estimated using the molecular mechanics combined with the generalized Born MM-GBSA along with the weighted interactions active-site residues (Miller et al., 2012). The binding free energy components can be represented according to the equations;

(1) $$△{\text{G}}_{\text{bind}}={\text{G}}_{\text{complex}}-\left(G_{\text{receptor}}+{\text{G}}_{\text{ligand}}\right)$$

(2) $$△{\text{G}}_{\text{bind}}=△\text{H}-\text{T}△\text{S}\sim △{\text{E}}_{\text{MM}}+△{\text{G}}_{\text{sol}}-\text{T}△\text{S}$$

(3) $$△{\text{E}}_{\text{MM}}=△{\text{E}}_{\text{inter}}+△{\text{E}}_{\text{electostatic}}+△{\text{E}}_{\text{vdw}}$$

(4) $$△{\text{E}}_{\text{sol}}=△{\text{G}}_{\text{GB}}+△{\text{G}}_{\text{SA}}$$

where, Δ*E*_*MM*_ represents the enthalpic components, whereas Δ*E*_*sol*_ represents the polar and nonpolar electrostatic components from solvation. Here, the polar electrostatic component is calculated using the GB model, whereas the nonpolar electrostatic contribution is calculated by SASA. The last 50-ns simulation trajectory is used, which was sampled per 10-ps interval.

## Data Availability Statement

The original contributions presented in the study are included in the article/Supplementary Material, further inquiries can be directed to the corresponding authors.

## Author Contributions

SK, PK, ND, GD, SR, and AP contributed to the conception, design of the study, and drafting of the article. SK and PK contributed to data generation and data analysis. All authors approved the final version of the article.

## Conflict of Interest

SK, GD, and ND were employed by the company miBiome Therapeutics LLP. The remaining authors declare that the research was conducted in the absence of any commercial or financial relationships that could be construed as a potential conflict of interest.
