# Supplementary material for: Combining SARS-CoV-2 Proofreading Exonuclease and RNA-Dependent RNA Polymerase Inhibitors as a Strategy to Combat COVID-19: A High-Throughput in silico Screening
Source: Front Microbiol. 2021 Jul 20;12:647693. doi: 10.3389/fmicb.2021.647693 (PMC8329495; doi:10.3389/fmicb.2021.647693)
Supplement: Supplementary file 2 [file Data_Sheet_2.PDF]

Supplementary Results:

RMSF analysis

To examine the conformational adaptability of Hesperidin and Glycyrrhizic acid during the simulation, we performed the RMSF analyses. RMSF defines the average positional fluctuations of the protein residues from their initial position which is important to determine the local dynamics of a protein. It also provides an important clue about the contribution of amino-acid residues involve in molecular interactions of protein-ligand for the spatial stability. In general, the residues belonging to stable secondary conformations (α-helix and β-sheet) show lower degree of residual fluctuations, whereas the residues belonging to terminal (N-and C-terminal) and loop regions have high degree of fluctuations. The three-dimensional structure of ExoN consists of seven α-helices (Arg76-His81, Trp159-Lys175, His188-Lys196, Leu253-His260, Ala270-Val287, Glu301-Asp324 and Arg485-Ala554) and fourteen β-sheets (Trp86-His95, Gln108-Gly110, Leu117-Val120, Gly123-Asp126, Val181-Leu185, Phe240-Ile242, Val328-Ile332, Glu348-Asp352, Gln364-Leu366, Val381-Trp384, Ser396-Tyr420, Phe427-Thr429, Ser507-Val510). Results of C^{α}-atoms RMSF shows that the residues of ExoN belonging to α-helices and β-sheets having average fluctuations <3.0 Å, whereas the residues of loops and N-and C-terminal are represented by average fluctuations >3.0 Å (**Supplementary Figure S2**).

In the supplementary figure S2, we can see that the average RMSF peaks of all the amino acids of ExoN-Hesperidin complex remain less than ExoN. Although the residual fluctuations of ExoN-Conivaptan complex are less as compare to ExoN, but, the residues at N-terminal and the region between β8-loop-β9 show relatively higher RMSF peaks. However, these residues are not involved in molecular interactions with drug molecules and placed away 20 Å from the active site. It may be possible that due the presence of longer loop between the structure of β8 and β9, under the sheer stress stable conformation of β-sheets show higher fluctuations. Thus, RMSF plots provide the structural evidence of stable molecular interaction of Hesperidin and Conivaptan with ExoN, respectively.

Essential dynamics (ED)

To obtain the dynamic picture of integrative changes on the binding of drug molecules with ExoN, we performed essential dynamics analyses of docked complex structures using the eigenvalues corresponding to eigenvector from the covariance matrix of C^{α}-atom coordinates which represent the principal components (PCs) (David & Jacobs 2014; Prakash, Kumar, Lynn & Haque 2019). The projection of two PCs, PC1 and PC2 along the native trajectory describes the collective motion of protein during the simulation. The plots of ED analysis for ExoN-

Astemizole, ExoN-Hesperidin, ExoN-Glycyrrhizic acid and ExoN-Convipatan are shown in **Supplementary Figure S5 A-D**, respectively.

Results highlight the recurrent features of ExoN-Astemizole and ExoN-Glycyrrhizic acid complex (**Supplementary Figure 5A and C**). The two complex explore a wide range of conformation space during the initial part of the simulation (0-10000 frames). The phase transition shifted to stable conformation space after around 100 ns (10000 frames). The shift is correlated to movement of drug molecules out of the pocket. suggesting that ExoN alone explores a wide range of conformation space during the initial 0-50 ns. In contrast, the docked complex of ExoN with Hesperidin and Conivaptan (**Supplementary Figure 5B and 5D**) represent a coherent collective motion of phase transitions (**Supplementary Figure 5B and 5C**). Thus, ED analyses suggested the stable conformational dynamics of Exon complexed with Hesperidin and Conivaptan as compared to ExoN-Glycyrrhizic acid and ExoN-Astemizole complex.

Supplementary Figures:

```
Expect        Identities              Positives
0.0           501/527(95%)            520/527(98%)

Query  1      AENVTGLFKDCSKIITGLHPTQAPTHLSVDIKFKTEGLCVDIPGIPKDMTYRRLISMMGF  60
              AENVTGLFKDCSK+ITGLHPTQAPTHLSVD KFKTEGLCVDIPGIPKDMTYRRLISMMGF
Sbjct  1      AENVTGLFKDCSKVITGLHPTQAPTHLSVDTKFKTEGLCVDIPGIPKDMTYRRLISMMGF  60

Query  61     KMNYQVNGYPNMFITREEAIRHVRAWIGFDVEGCHATRDAVGTNLPLQLGFSTGVNLVAV  120
              KMNYQVNGYPNMFITREEAIRHVRAWIGFDVEGCHATR+AVGTNLPLQLGFSTGVNLVAV
Sbjct  61     KMNYQVNGYPNMFITREEAIRHVRAWIGFDVEGCHATREAVGTNLPLQLGFSTGVNLVAV  120

Query  121    PTGYVDTENNTEFTRVNAKPPPGDQFKHLIPLMYKGLPWNVVRIKIVQMLSDTLKGLSDR  180
              PTGYVDT NNT+F+RV+AKPPPGDQFKHLIPLMYKGLPWNVVRIKIVQMLSDTLK LSDR
Sbjct  121    PTGYVDTPNNTDFSRVSAKPPPGDQFKHLIPLMYKGLPWNVVRIKIVQMLSDTLKNLSDR  180

Query  181    VVFVLWAHGFELTSMKYFVKIGPERTCCLCDKRATCFSTSSDTYACWNHSVGFDYVYNPF  240
              VVFVLWAHGFELTSMKYFVKIGPERTCCLCD+RATCFST+SDTYACW+HS+GFDYVYNPF
Sbjct  181    VVFVLWAHGFELTSMKYFVKIGPERTCCLCDRRATCFSTASDTYACWHHSIGFDYVYNPF  240

Query  241    MIDVQQWGFTGNLQSNHDQHCQVHGNAHVASCDAIMTRCLAVHECFVKRVDWSVEYPIIG  300
              MIDVQQWGFTGNLQSNHD +CQVHGNAHVASCDAIMTRCLAVHECFVKRVDW++EYPIIG
Sbjct  241    MIDVQQWGFTGNLQSNHDLYCQVHGNAHVASCDAIMTRCLAVHECFVKRVDWTIEYPIIG  300

Query  301    DELRVNSACRKVQHMVVKSALLADKFPVLHDIGNPKAIKCVPQAEVEWKFYDAQPCSDKA  360
              DEL++N+ACRKVQHMVVK+ALLADKFPVLHDIGNPKAIKCVPQA+VEWKFYDAQPCSDKA
Sbjct  301    DELKINAACRKVQHMVVKAALLADKFPVLHDIGNPKAIKCVPQADVEWKFYDAQPCSDKA  360

Query  361    YKIEELFYSYATHHDKFTDGVCLFWNCNVDRYPANAIVCRFDTRVLSNLNLPGCDGGSLY  420
              YKIEELFYSYATH DKFTDGVCLFWNCNVDRYPAN+IVCRFDTRVLSNLNLPGCDGGSLY
Sbjct  361    YKIEELFYSYATHSDKFTDGVCLFWNCNVDRYPANSIVCRFDTRVLSNLNLPGCDGGSLY  420

Query  421    VNKHAFHTPAFDKSAFTNLKQLPFFYYSDSPCESHGKQVVSDIDYVPLKSATCITRCNLG  480
              VNKHAFHTPAFDKSAF NLKQLPFFYYSDSPCESHGKQVVSDIDYVPLKSATCITRCNLG
Sbjct  421    VNKHAFHTPAFDKSAFVNLKQLPFFYYSDSPCESHGKQVVSDIDYVPLKSATCITRCNLG  480

Query  481    GAVCRHHANEYRQYLDAYNMMISAGFSLWIYKQFDTYNLWNTFTRLQ  527
              GAVCRHHANEYR YLDAYNMMISAGFSLW+YKQFDTYNLWNTFTRLQ
Sbjct  481    GAVCRHHANEYRLYLDAYNMMISAGFSLWVYKQFDTYNLWNTFTRLQ  527
```

Supplementary Figure S1: Pairwise alignment of nsp14 from SARS-CoV and SARS-CoV2 Alignment of SARS-CoV nsp14 (Query) and SARS-CoV2 nsp14 (Subject) to depict conservation of catalytic residues (highlighted in green). Arrow depicts end of ExoN domain and start of methyl transferase domain.

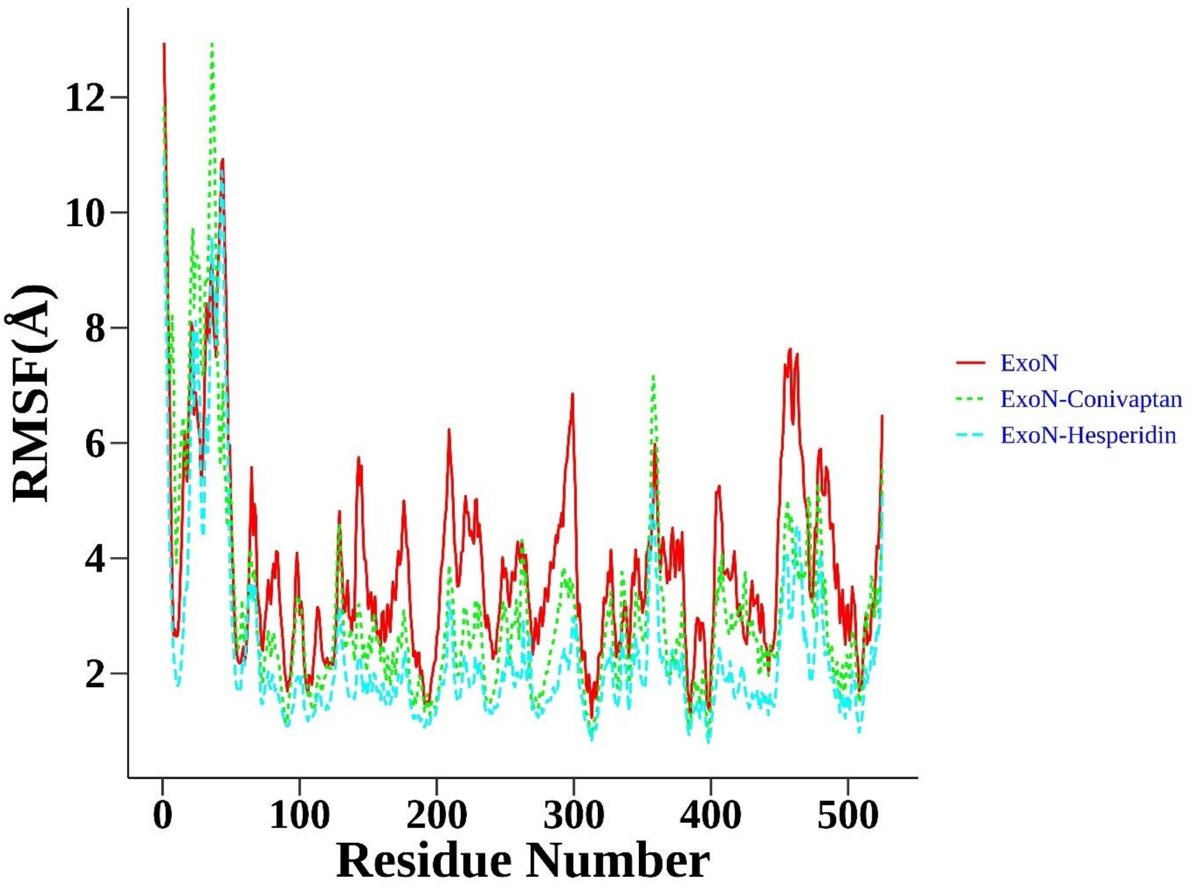

Supplementary Figure S2: RMSF plot of all Cα-atoms of Exon, ExoN-Conivaptan and ExoN-Hesperidin.

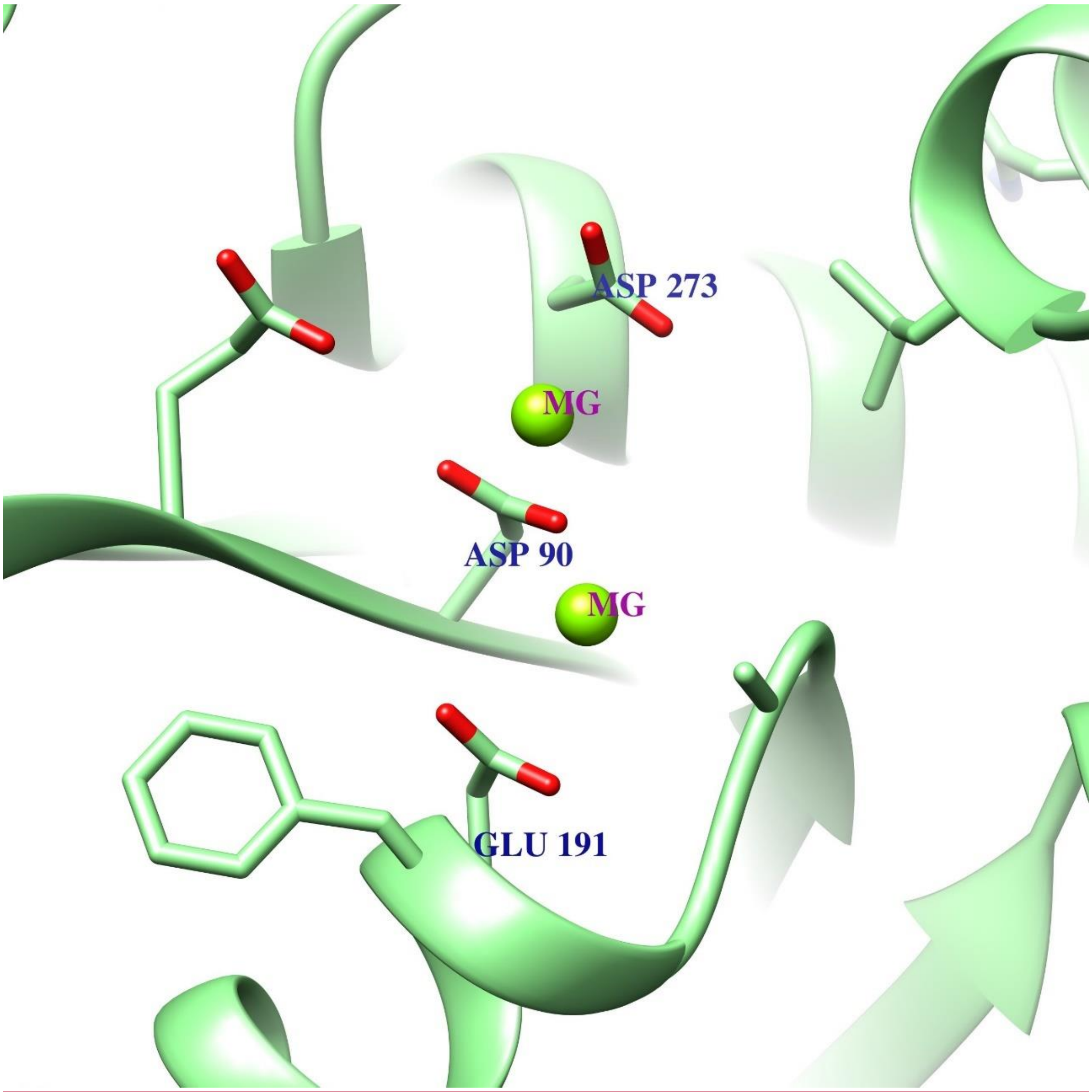

Supplementary Figure S3: Catalytic site of ExoN containing two Mg^{2+} ion. Average distance of metal ions in four complexes remain consistent around ~3.6 Å.

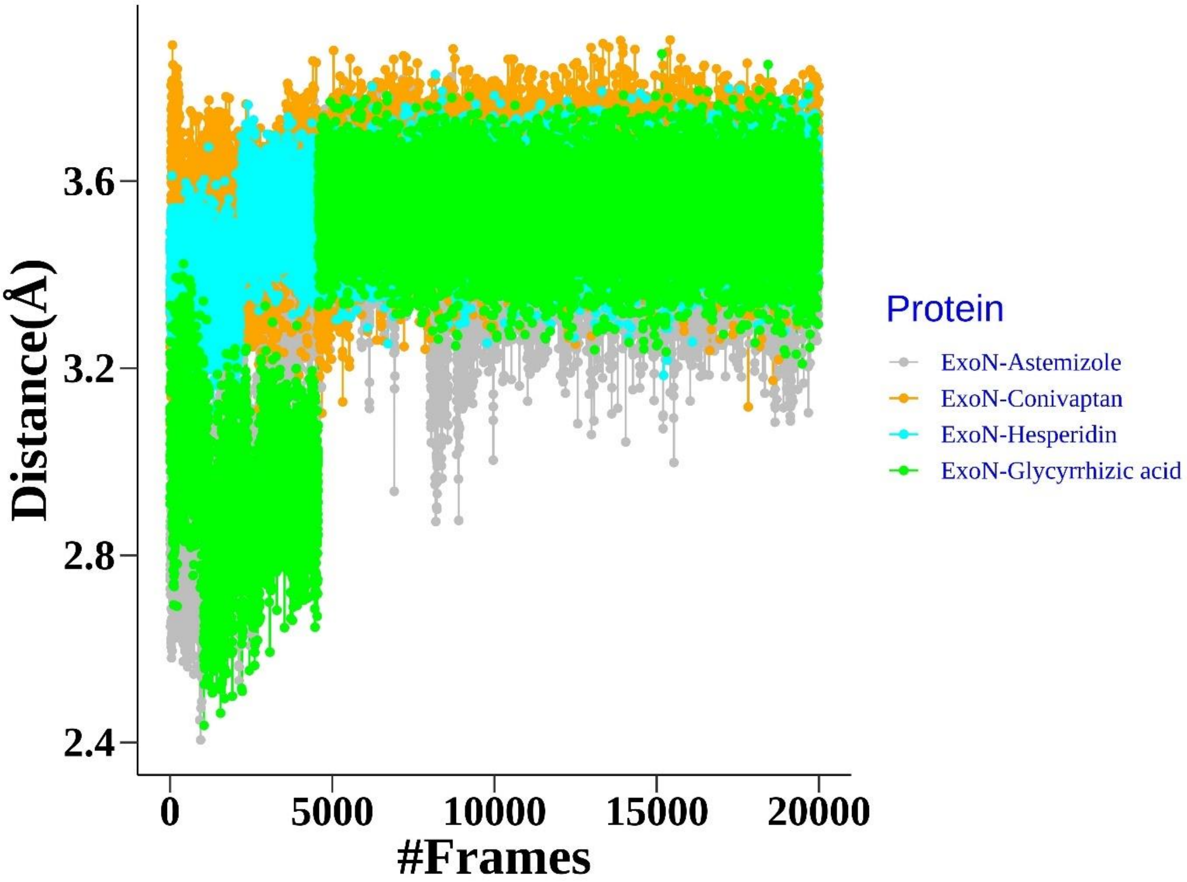

Supplementary Figure S4: Average distance of metal ions (two Mg^{2+}) in four complexes remain constant at around ~3.6 Å.

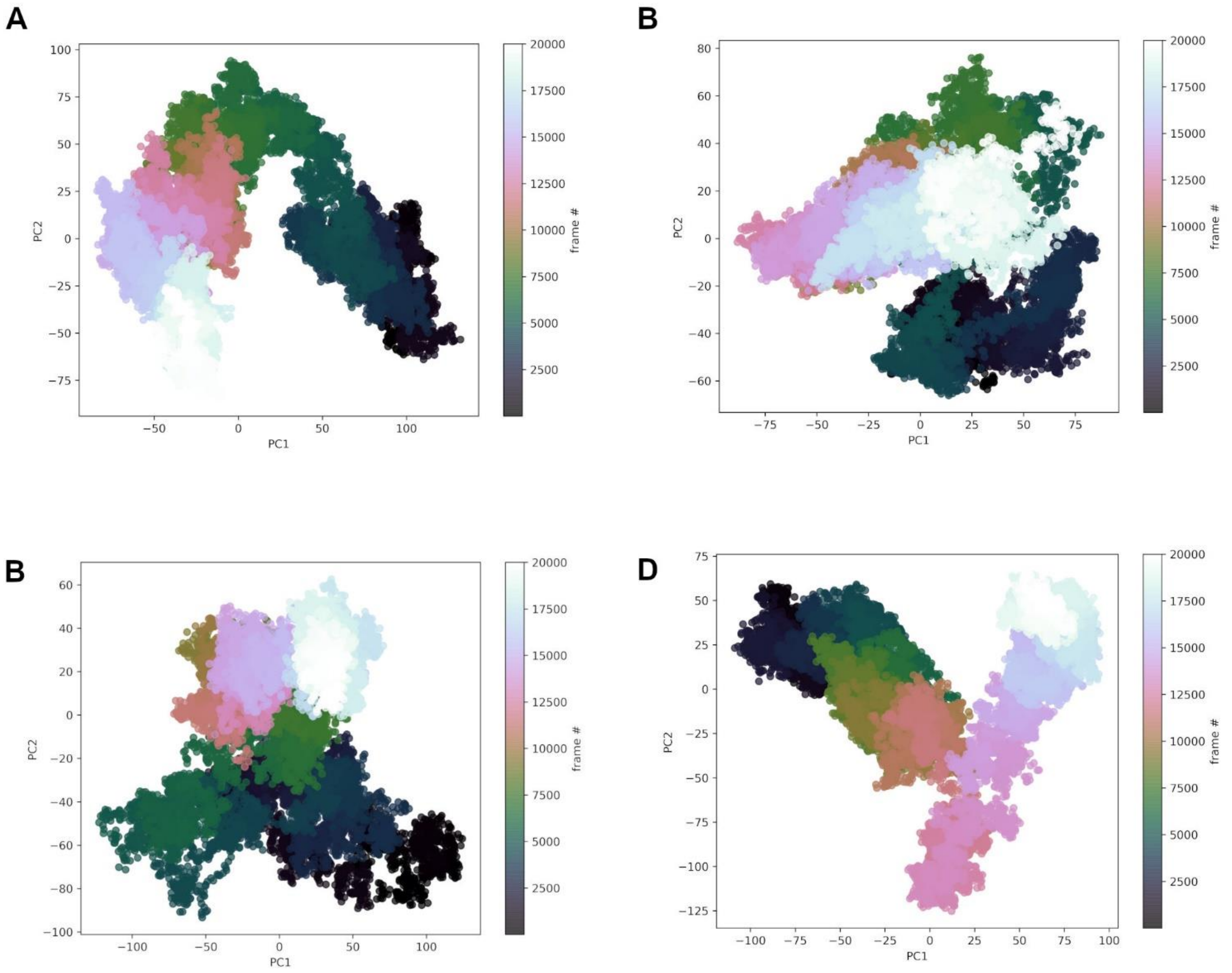

Supplementary Figure S5. Essential dynamics plots of (A) ExoN-Astemizole, (B) ExoN-Hesperidin, (C) ExoN-Glycyrrhizic acid and (D) ExoN-Conivaptan acid. The collective motion of protein is represented according time evolution, defined in the right panel of each plot in nanoseconds (ns).

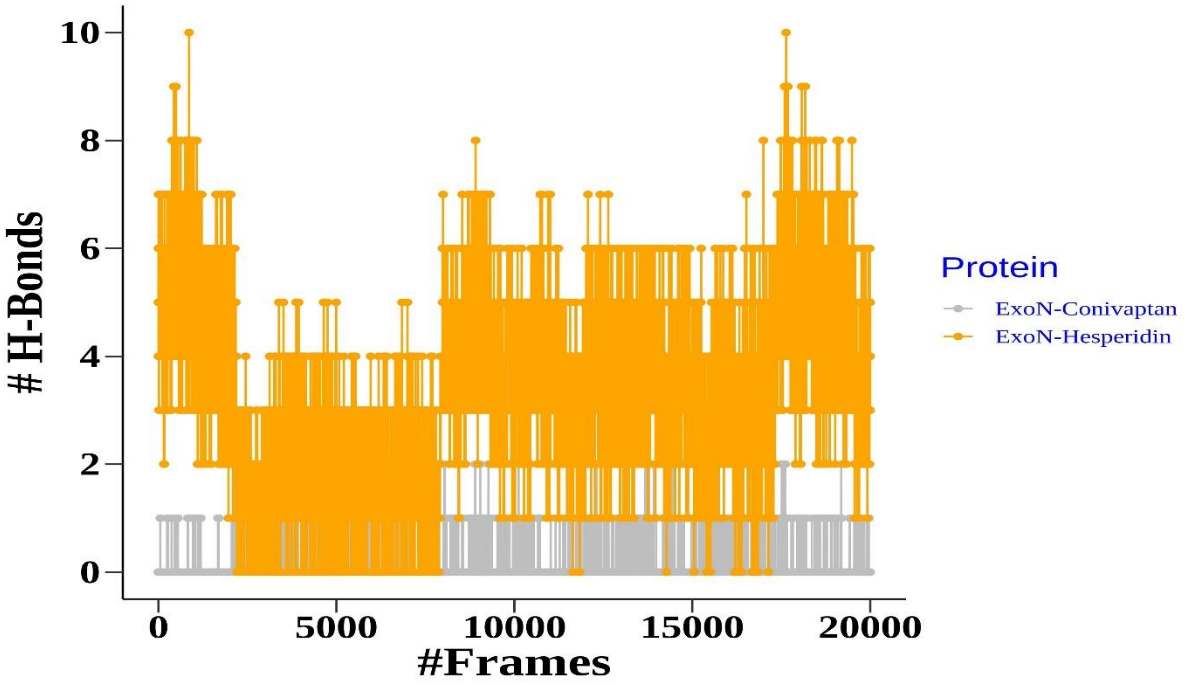

Supplementary Figure S5. The time evolution plots of hydrogen-bonds (H-bonds) between the ExoN and drug molecules Conivaptan and Hesperidin, during the simulation period of 200 ns.
